# Comparison of Effects of an Endotracheal Tube or Facemask on Breathing Pattern and Distribution of Ventilation in Anesthetized Horses

**DOI:** 10.3389/fvets.2022.895268

**Published:** 2022-06-09

**Authors:** Fernando Moreno-Martinez, David Byrne, Anthea Raisis, Andreas D. Waldmann, Giselle Hosgood, Martina Mosing

**Affiliations:** ^1^College of Veterinary Medicine, Murdoch University, Perth, WA, Australia; ^2^Department of Anaesthesiology and Intensive Care Medicine, Rostock University Medical Centre, Rostock, Germany

**Keywords:** electric impedance tomography, distribution of ventilation, *triple drip*, endotracheal tube (ETT), sigh, venous admixture, *crown-like*

## Abstract

Equine respiratory physiology might be influenced by the presence of an endotracheal tube (ETT). This experimental, randomized cross-over study aimed to compare breathing pattern (BrP) and ventilation distribution in anesthetized horses spontaneously breathing room air via ETT or facemask (MASK). Six healthy adult horses were anesthetized with total intravenous anesthesia (TIVA; xylazine, ketamine, guaiphenesin), breathing spontaneously in right lateral recumbency, and randomly assigned to ETT or MASK for 30 min, followed by the other treatment for an additional 30 min. During a second anesthesia 1 month later, the treatment order was inversed. Electrical impedance tomography (EIT) using a thoracic electrode belt, spirometry, volumetric capnography, esophageal pressure difference (ΔPoes), venous admixture, and laryngoscopy data were recorded over 2 min every 15 min. Breaths were classified as normal or alternate (sigh or *crown-like*) according to the EIT impedance curve. A mixed linear model was used to test the effect of treatment on continuous outcomes. Cochran-Mantel-Haenszel analysis was used to test for associations between global BrP and treatment. Global BrP was associated with treatment (*p* = 0.012) with more alternate breaths during ETT. The center of ventilation right-to-left (CoV_RL_) showed more ventilation in the non-dependent lung during ETT (*p* = 0.025). The I:E ratio (*p* = 0.017) and ΔPoes (*p* < 0.001) were smaller, and peak expiratory flow (*p* = 0.009) and physiologic dead space (*p* = 0.034) were larger with ETT. The presence of an ETT alters BrP and shifts ventilation toward the non-dependent lung in spontaneously breathing horses anesthetized with TIVA.

## Introduction

Inspiration and expiration in most mammals are monophasic; however, the breathing pattern (BrP) in horses is biphasic, with both active and passive inspiration and expiration (1, 2). Equine BrP can change during physiologically challenging situations such as swimming or during and after general anesthesia, especially when expiratory gas flow patterns might be altered by laryngeal closure or diaphragmatic breaking which results in breath-holding (3–5).

In most studies describing BrP in anesthetized horses, the animals were orotracheally intubated, which would impair physiologic laryngeal movements and therefore impact the horse's ability to modify its BrP (6, 7). The endotracheal tube (ETT) also reduces the work of breathing in the anesthetized, spontaneously-breathing horse because it reduces upper airway respiratory resistance. This reduction in airway resistance impacts respiratory mechanics and can influence BrP (8, 9).

Electrical impedance tomography (EIT) is a non-invasive imaging technology that measures changes in thoracic resistance over time on a breath-by-breath basis (10). By applying alternating current through an EIT electrode-belt positioned around the thorax, impedance changes (ΔZ) can be measured and cross-sectional 2D images of the thorax are generated. With EIT, events such as inspiratory breath-holding as well as the distribution of ventilation can be evaluated in awake and anesthetized horses on the images using reconstruction algorithms (4, 11, 12).

Based on the assumption that ETT impedes laryngeal function and alters respiratory mechanics, our study aimed to compare breathing patterns and ventilation distribution using EIT in anesthetized horses allowed to spontaneously breathe room air via ETT or facemask (MASK). Our hypothesis was that breathing patterns and distribution of ventilation would differ between these two forms of airway management.

## Materials and Methods

Ethics approval was obtained by the Animal Ethics Committee at Murdoch University (R3294/20). Each horse was anesthetized twice for this experimental, randomized cross-over study. Animals were allowed to breathe room air spontaneously via ETT or MASK, considered the treatment, for 30 min, followed by the other treatment for an additional 30 min. A coin was used to assign the randomized treatment order for the first anesthesia. During a second anesthesia 1 month later, the treatment order was inversed. The study design aimed to minimize the effect of variation and maximize the gain from a small sample size. A sample size of six horses in this paired design would allow detection of a minimum effect size of 1.4.

### Animals

Six male horses (Standardbred *n* = 4, Thoroughbred *n* = 2), were recruited from the Murdoch teaching herd with a median age of 13 years (range 8–21years), a median bodyweight of 553 kg (range 487–634 kg), and a median BCS of 5/9 (range 4–7/9). Horses were clinically healthy (ASA I) as per history and clinical exam.

Horses were moved from the teaching herd into irrigated paddocks in pairs at least 48 h before the experiment to which they were previously acclimatized. These paddocks offered unrestricted access to Kikuyu grass and water. While in the paddock, horses also received supplemental feed of oaten hay.

### Pre-anesthetic Preparation and Anesthesia

An arterial blood sample was collected from the transverse facial artery for confirmation of adequate gas exchange with the partial pressure of oxygen (PaO_2_) >90 mmHg and carbon dioxide (PaCO_2_) <45 mmHg 24 h prior to anesthesia. On the day of the experiment, the horse was moved into stocks and a 14-gauge catheter (BD Angiocath IV Catheter, BD ANZ, Melbourne, Australia) was placed aseptically in the right jugular vein after local infiltration block of lidocaine (Lignocaine Ilium, Troy Laboratories Pty Limited, Australia) in the right jugular vein. Xylazine 0.5 mg kg^1^ (Ilium Xylazil-100, Troy Animal Healthcare, Glendenning NSW, Australia) was administered intravenously (IV). After placing another 14-gauge catheter aseptically in the left jugular vein, a 90 cm-long 19-gauge catheter (MILA long line catheter, MILA International, Florence, KY, USA) was introduced with its tip located in the pulmonary artery, verified using pressure waveforms. For esophageal pressure measurement, a rigid polyamide 220 cm-long, 6 mm-diameter pressure tube with a thin-walled latex balloon fixed at the tip was threaded through a nasogastric tube. The complex was advanced through the left nostril of the horse into the esophagus. The nasogastric tube was removed, leaving the tip of the balloon at the level of the 5th intercostal space based on a premeasured mark on the rigid tube.

The horse's thorax was wetted with tap water and a customized EIT belt (32 gold-plated washer electrodes, 1 plane) placed at the level of the 6th intercostal space, held in contact with the skin by application of a surrounding cohesive bandage (13).

The horse was moved into the induction box and additional xylazine 0.7 mg kg^−1^ IV was administered. Three min later, anesthesia was induced with ketamine (Ketamine Ceva, Ceva Animal Health Pty Ltd, Australia) 2 mg kg^−1^ IV and diazepam (Diazepam Ilium, Troy Laboratories Pty limited, Australia) 0.1 mg kg^−1^ IV. Once recumbent, the horse was hoisted onto a padded surgical table and positioned in right lateral recumbency.

Anesthesia was maintained with an IV infusion of *triple drip* using xylazine 1 mg kg^−1^ h^−1^, ketamine 2 mg kg^−1^ h^−1^, and guaiphenesin (Guaiphenesin Ilium, Troy Laboratories Pty limited, Australia) 75 mg kg^−1^ h^−1^; equivalent to 0.78 ml kg^−1^ h^−1^ of the drug mixture. For the first 15 min, doses were doubled. Anesthetic depth was monitored constantly by an experienced anesthetist (AR) and additional xylazine 0.25 mg kg^−1^ IV and ketamine 0.5 mg kg^−1^ IV were administered to increase the depth of anesthesia if required. Hartmann's solution 3 mL kg^−1^ h^−1^ IV (Hartmann's solution, Baxter Healthcare Pty Ltd, Australia) was administered through the right jugular catheter. A urinary catheter (Foley 24Fr 30cc, MILA International Inc, KY, US) was placed to allow free flow of urine. Monitoring beside the one specified below consisted of a lead II electrocardiogram (SurgiVet^®^ Smith Medicals, Minnesota USA) and pulse-oximetry (Radical-7^®^ Masimo Corporation^®^, Irvine CA). Heart rate, respiratory rate, partial pressure of end-tidal CO_2_, systolic, mean, and diastolic arterial pressures, without a, just saturation of hemoglobin, and signs of the depth of anesthesia were manually recorded every 5 min for the duration of anesthesia.

An arterial catheter was placed aseptically for invasive blood pressure measurement and blood sampling in the left metatarsal artery. The catheter was connected to a pressure transducer (Meritrans DTX Plus, MeritMedical, UT, US) zeroed to atmospheric pressure at the level of the sternum.

Horses breathed room air spontaneously for the duration of anesthesia. Oxygen supplementation was provided when two consecutive PaO_2_ measurements taken 15 min apart were below 60 mmHg. Oxygen supplementation was delivered at 10–15 L/min through the ETT or MASK via a customized large animal non-rebreathing system (14).

After 60 min of anesthesia, *triple drip* infusion was discontinued, the EIT belt and all catheters removed, and the horse hoisted to a padded recovery box for unassisted recovery. Fifteen min after standing, the horses were walked back to the paddock and observed for adverse events for another 24 h before returning to the herd.

### Intra-Anesthesia Instrumentation

**Figure 1:**
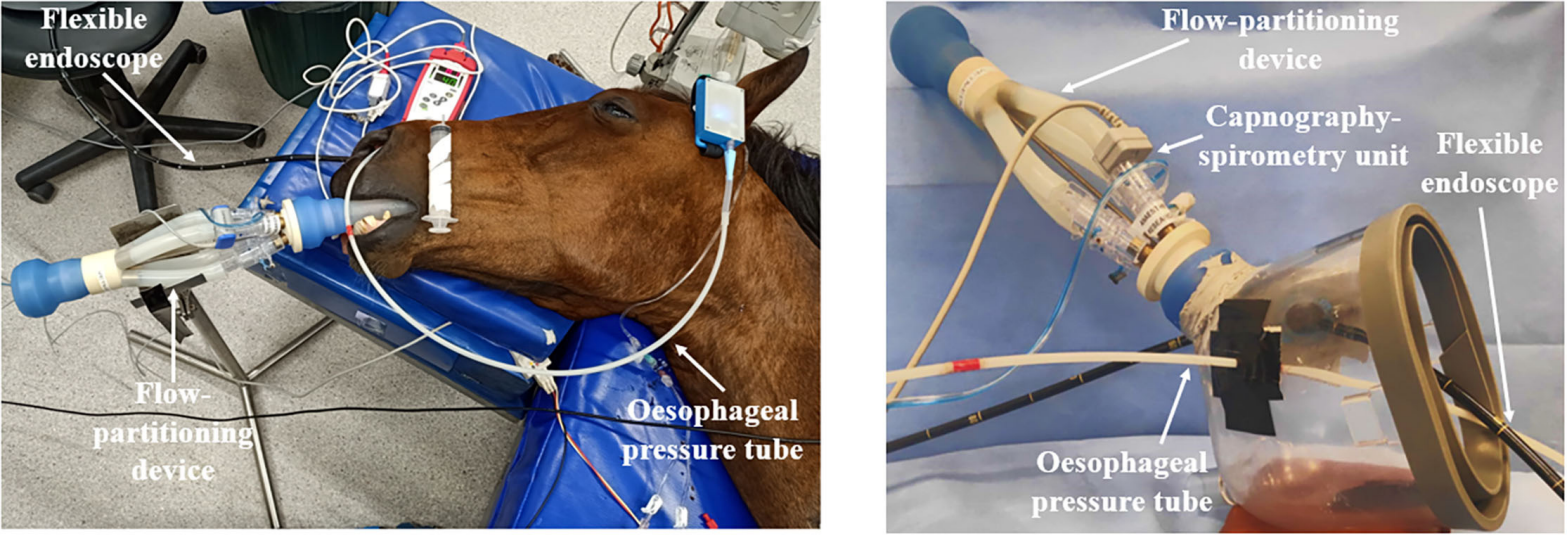
Set-up with spirometer, volumetric capnography, esophageal pressure device, and flexible endoscope for an endotracheally intubated horse (left) and for the use of a facemask (right).

Once the horse was anesthetized and positioned on the table (T0), either orotracheal intubation was performed using a 26 mm ID ETT (ETT treatment) or a tight-fitting facemask was placed over their muzzle and nostrils (MASK treatment) according to the order of assigned treatment. The facemask was modified with two elastic rubber seals to pass the esophageal tube and a flexible endoscope without creating significant leaks (Figure 1).

The position of the EIT belt was checked after being positioned on the table. The EIT electrode belt was connected to the EIT device (BBVet, SenTec AG, EIT branch, Switzerland), linked to a laptop with dedicated software to record EIT data applying an equine species-specific finite element model (BBVet SW, SenTec, Switzerland).

A flow-partitioning device (FPD) that divides the inspiratory and expiratory flow into four equal parts was attached to the silicon adaptor of the ETT or MASK (Figure 1) (15). A human spirometer and mainstream capnograph (FluxmedView, MBMED, Argentina) were fitted to the upper partition for spirometry and volumetric capnography (VCap) measurements. The capnograph and spirometer were calibrated according to the manufacturer's recommendation before each use. The accuracy of the FDP-spirometer combination was checked with a 3 L syringe (Model 5,630 series, Hans Rudolph Inc., Shawnee, KS, USA) for volumes between 1, 1.5, 2, and 3 L over 3 measurements before the start of the experiment. The overall bias ± standard deviation was 5.79% ± 3.08%. Leaks in the system were determined by visual inspection of the flow-volume loop of the spirometer software. Where the loop did not close, indicating a leak, the ETT cuff was further inflated, or cotton wool was placed around the mask and muzzle to create an airtight seal.

The esophageal tube was connected to a portable pressure measurement device (Figure 1) (Ventiplot dppl measurement device, JP. Schramel, Vetmeduni Vienna, Austria) calibrated to atmospheric pressure as per manufacturer's guidelines. Esophageal pressure was displayed on a tablet (Bluefruit Connect, Adafruit Industries, NY, US).

A 180 cm flexible endoscope (Tele Pak Vet X LED with 6011PKSK/NKSK, KARL STORZ Endoscopy Australia Pty. Ltd., Macquarie Park NSW, Australia) was passed through the right nostril and advanced to visualize the larynx (Figure 1).

The location of the tip of the pulmonary artery catheter was re-confirmed by pressure measurement to rule out potential dislodgement during induction and hoisting.

### Study Timeline and Data Collection

**Figure 2:**
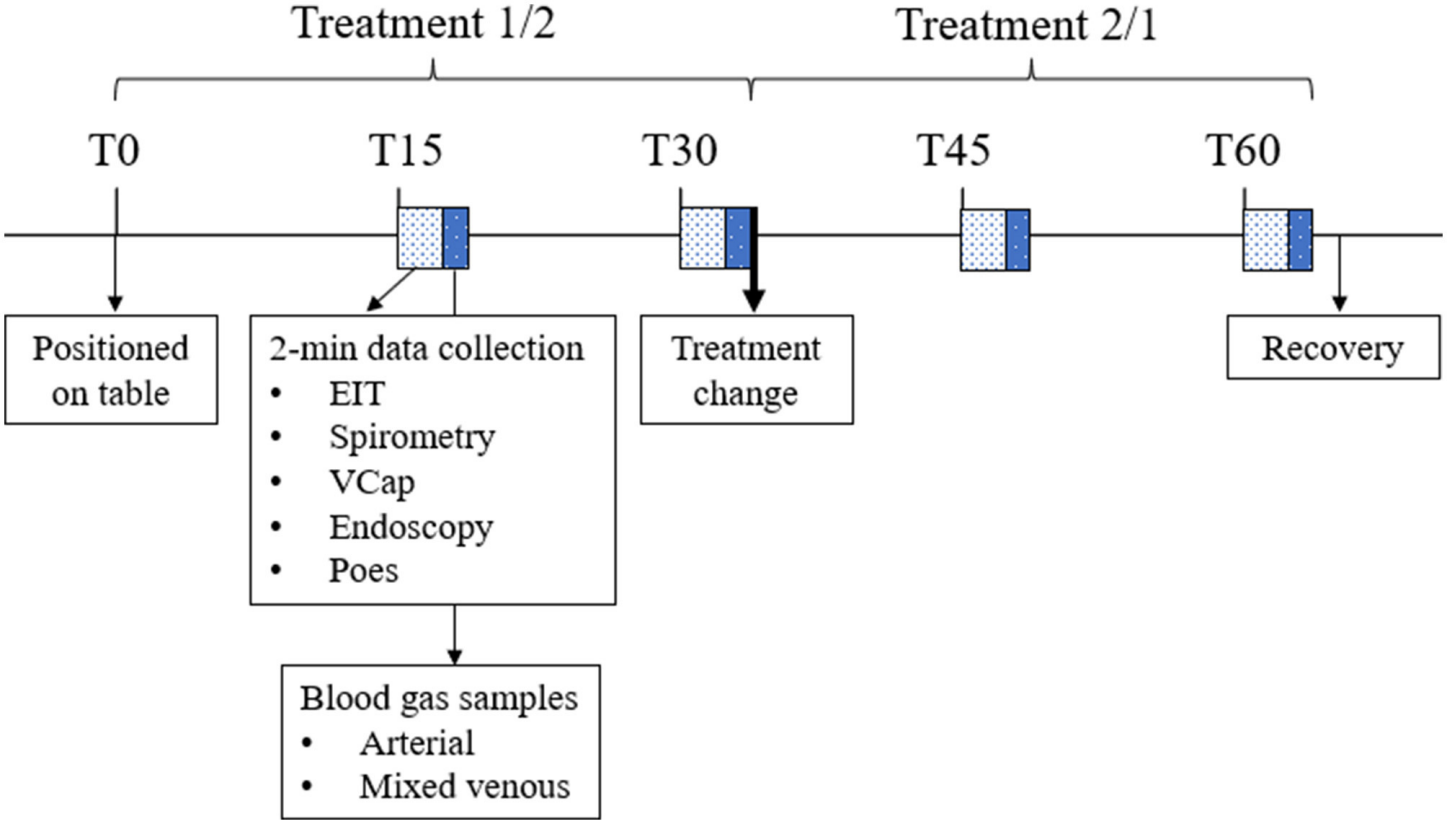
Timeline of the experiment showing the interventions and data collection throughout one anesthesia. EIT, electrical impedance tomography data; Vcap, volumetric capnography; Poes, esophageal pressure. During the first half of the experiment, one treatment was applied (either ETT or MASK). After data collection at T30, treatment was changed. Horses were allowed to recover from general anesthesia after data collection at T60.

Data were collected at 15 (T15), 30 (T30), 45 (T45), and 60 (T60) min after positioning the horse on the table (T0). After data collection at T30, horses assigned ETT were extubated and the face mask positioned over the muzzle, while horses assigned MASK had the facemask removed and were orotracheally intubated (Figure 2).

At each time point EIT, spirometry, VCap, and endoscopy data were recorded over 2 min. Over the space of these 2 min, three paired (maximum-expiratory and minimum-inspiratory) esophageal pressure values were manually recorded.

After each 2-min recording period, arterial and mixed venous blood samples were collected simultaneously and analyzed immediately for partial pressure of oxygen (PaO_2_ and P$\overset{-}{\text{v}}$O_2_) and carbon dioxide (PaCO_2_ and P$\overset{-}{\text{v}}$CO_2_) and hemoglobin concentration (Radiometer ABL800 FLEX, Radiometer Pacify Pty. Ltd., Australia). Atmospheric pressure on the day of the experiment was recorded (http://www.bom.gov.au/wa/observations/perth.shtml). Inspiratory oxygen fraction (FiO_2_) was measured via an adaptor in the T-piece of the non-rebreathing system and recorded if different from 0.21.

One video camera continuously recorded all monitors, and a second camera recorded the horse's thorax and abdomen during the duration of anesthesia for retrospective synchronization of monitor data with observed breathing movements.

### Data Analysis

#### EIT Data

##### Pilot Study on Breathing Pattern

Before commencing the study described here, three horses were anesthetized using the same protocol in the field for student teaching with the EIT electrode belt in place (ethics approval number: R3272/20). EIT data were recorded after orotracheal intubation and during periods of unsecured airway.

**Figure 3:**
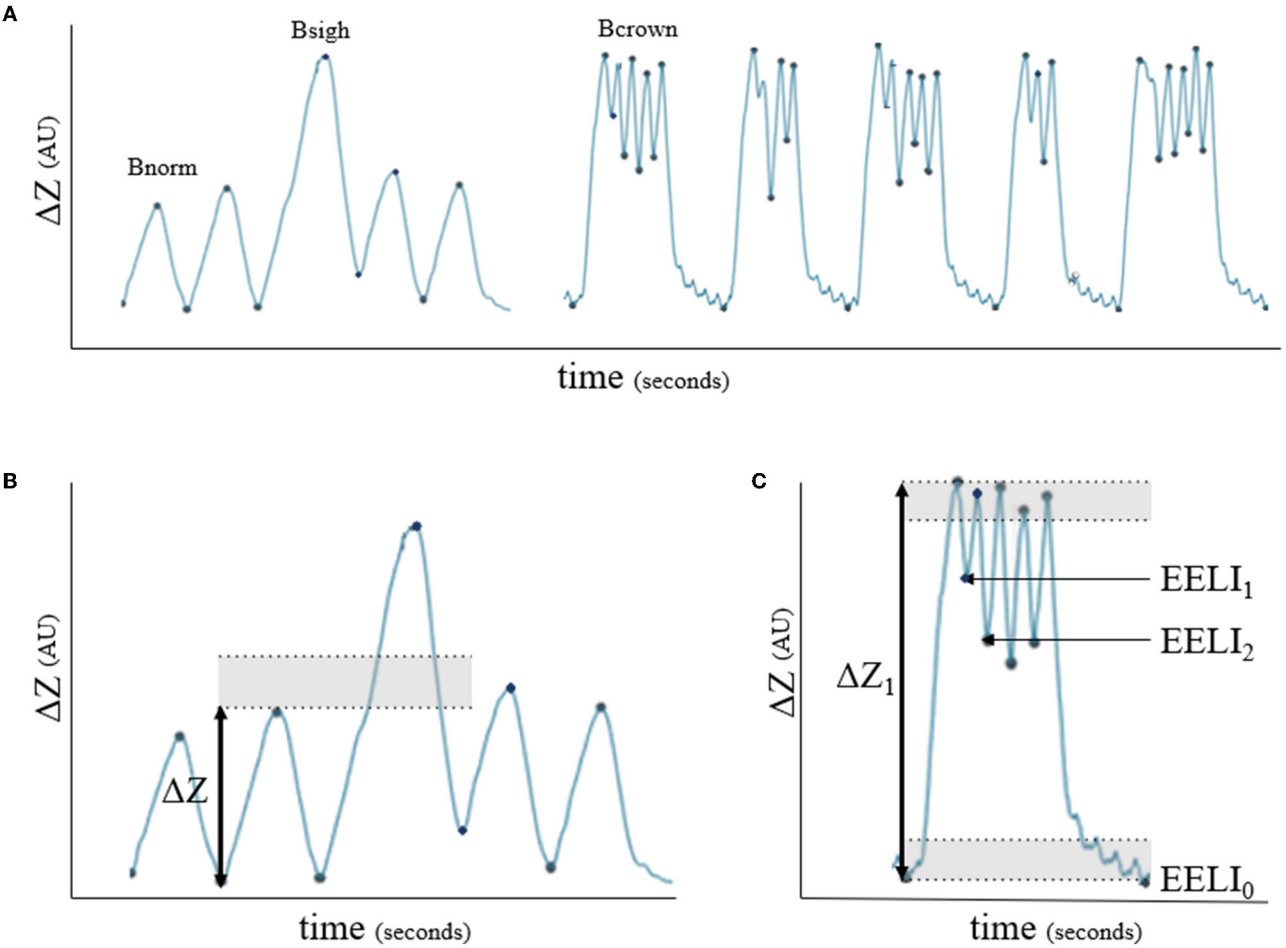
Impedance curves (ΔZ, arbitrary units) for breath classification. **(A)** Bnorm: normal breath with linear inspiration and either linear or concave expiration; Bsigh: breath showing >1.3 times ΔZ of a previous breath; Bcrown: group of breaths showing incomplete expiration in between where the end-expiratory lung impedance (EELI) does not return to baseline between breaths. **(B)** Example of Bsigh: shaded area represents 1.3 times ΔZ. **(C)** Example of Bcrown: shaded area indicates 10% of the first breath's ΔZ (ΔZ_1_) which was defined as baseline EELI (EELI_0_) for each breath. If the start of the following breath did not appear in the shaded area (EELI_1_ and EELI_2_), the breaths were classified as Bcrown.

During direct observation of the respiratory movements, different breath patterns were evident; horses showed intermittent deep breaths and clusters of breaths with incomplete expiration between breaths. Retrospective analysis of the EIT impedance curves of these horses suggested that the alternate deep breaths caused an increase in impedance change of at least 30% compared to the previous breath. The impedance curve recorded during the cluster of breaths showed a *crown-like* shape, where the end-expiratory lung impedance (EELI) between breaths remained higher than the EELI from the first and final breath of the cluster (Figure 3).

##### EIT Data and Breathing Pattern

EIT data for each 2-min recording was analyzed using a dedicated EIT software (Ibex, Sentec AG, EIT branch, Landquart, Switzerland). Breath-by-breath measurements were averaged for each variable at the relevant time points and transferred to a spreadsheet for statistical analysis.

**Figure 4:**
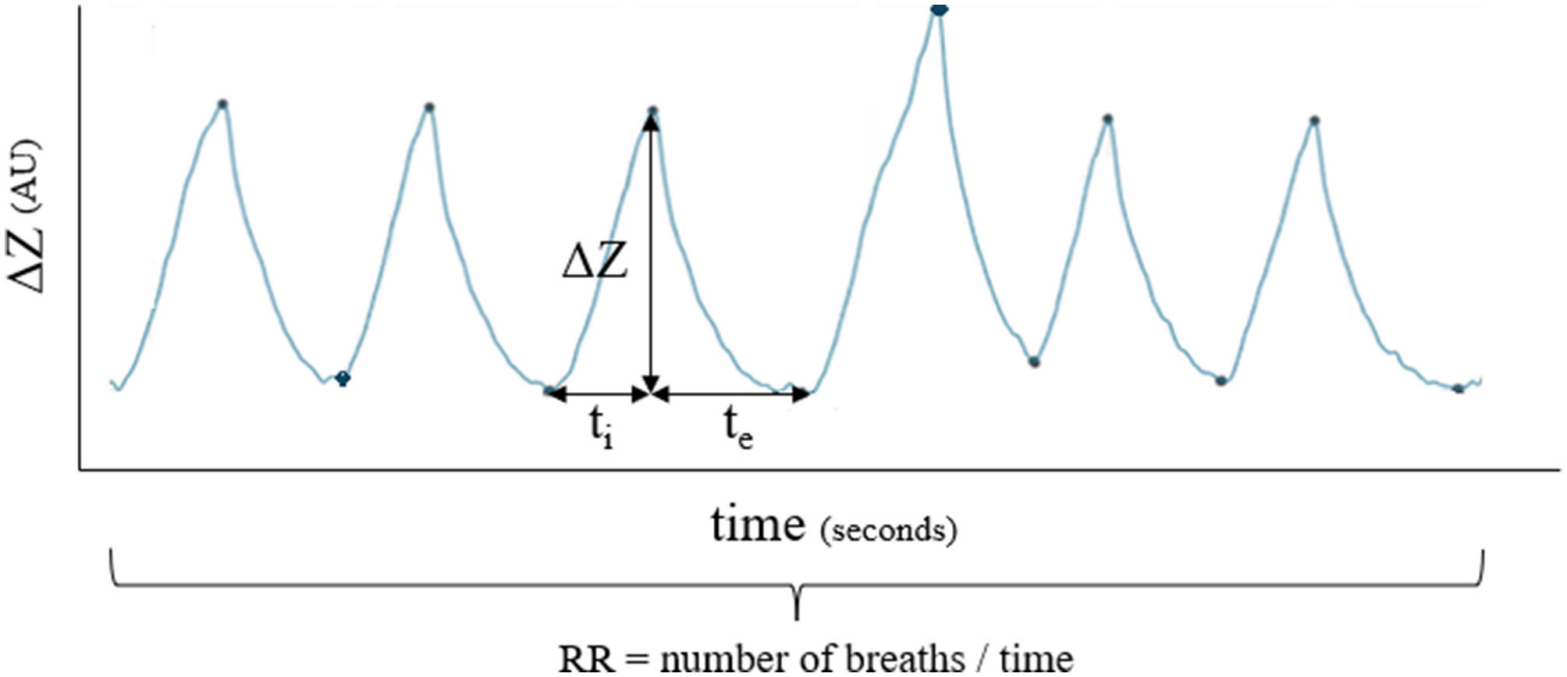
Example of how respiratory rate (RR), inspiratory time (t_i_) and expiratory time (t_e_) were calculated for each breath.

Respiratory rate, inspiratory, and expiratory time were extracted from the impedance curve (Figure 4). Inspiratory time (t_i_) was calculated from the start of the impedance change to the peak of impedance change. Expiratory time (t_e_) was calculated from the peak of impedance change to the start of the next inspiration. The inspiratory to expiratory (I: E) ratio was calculated.

Each breath within the measurement periods was classified as normal or alternate (*sigh* or *crown-like*) (Figure 3) based on the review of EIT impedance curves and video recordings of respiratory excursions.

The following definitions based on the pilot study were used for classification:

- Normal breath (Bnorm): a linear, uninterrupted inspiration immediately followed by a linear or concave full expiration back to the previous EELI (4).
- *Sigh* breath (Bsigh): a breath with the same shape of the impedance curve as Bnorm but where ΔZ was at least 1.3 times the ΔZ of the previous breath.
- *Crown-like* breath (Bcrown): a group of breaths with failure to return to the end expiratory lung impedance between breaths.

After classifying each breath, a global breathing pattern (gBrP) was assigned per measurement timepoint as: normal (Bnorm for all breaths) or alternate (one or more Bsigh or Bcrown).

##### Distribution of Ventilation

Ten to twenty consecutive artifact-free breaths, representative for the measurement period, were selected for further analysis. Each of the breaths within a *crown-like* pattern was analyzed as independent breaths. The following EIT variables were analyzed per breath:

- Center of ventilation (CoV) from ventral to dorsal (CoV_VD_) and from right to left (CoV_RL_). A CoV expresses the geometric focal point of overall ventilation as a single value, where 0% represents the ventral or right edge of the EIT image and 100% represents the dorsal or left edge for CoV_VD_ and COV_RL_, respectively.
- Regional ΔZ as described by the regions in an equine finite element model. There are eight regions, four in the left (L) and four in the right (R) lung. Within each lung, regions are vertically stacked comprising 25% of the vertical height: dorsal (D), central-dorsal (CD), central-ventral (CV), and ventral (V). Therefore, all the regional variables calculated as percentages of the total ΔZ were ΔZ_L_ and ΔZ_R_ for each of the lungs, and ΔZ_L_D__, ΔZ_L_CD__, ΔZ_L_VC__, ΔZ_L_V__ (left) and ΔZ_R_D__, ΔZ_R_CD__, ΔZ_R_CV__ and ΔZ_R_V__ (right).
- Dependent and non-dependent silent spaces (DSS and NSS, respectively). Silent spaces include areas within the lung region of interest (ROI) that show a change in impedance <10% of the maximal ΔZ (16). Silent spaces are expressed as a percentage of the total area of the lung and represent hypoventilated areas.
- Tidal impedance variation (TIV; arbitrary units). TIV represents the total ΔZ over one breath and can be used as the EIT surrogate of tidal volume in large animals (17, 18).

#### Spirometry and Volumetric Capnography

Spirometry and volumetric capnography data were analyzed using dedicated software (FluxReviewGrTCO2, MBMED, Argentina).

Data extracted for analysis were indexed tidal volume (V_T_·kg^−1^), indexed alveolar tidal volume (V_Talv_·kg^−1^), indexed minute ventilation (MV·kg^−1^), peak inspiratory and expiratory flow (PIF and PEF, respectively), EtCO_2_, indexed elimination of CO_2_ (VCO_2_·kg^−1^) and airway dead-space (V_Daw_/V_T_). Physiological dead-space (V_Dphys_/V_T_) was calculated by the software based on Bohr's equation (19).

#### Esophageal Pressure

From the three paired recorded minimum and maximum esophageal pressure values, the difference (ΔPoes) was calculated, and the mean difference was used for statistical analysis.

#### Blood Sampling and Gas Analysis

Measurements of PaO_2_ and P$\overset{-}{\text{v}}$O_2_ were used to calculate equine-specific arterial and mixed venous hemoglobin saturation (20). Venous admixture $\left({\dot{\text{Q}}}_{\text{s}}/{\dot{\text{Q}}}_{\text{t}}\right)$ was calculated using Berggren's standard equation (21). PaO_2_, PaCO_2_, and ${\dot{\text{Q}}}_{\text{s}}/{\dot{\text{Q}}}_{\text{t}}$ were included in the statistical analysis.

#### Endoscopy

The expiratory movement of the arytenoid cartilages was subjectively scored during MASK treatment from 1 to 3 being: 1, no movement; 2, partial adduction; 3, full glottic closure (arytenoids touching each other).

#### Statistics

All continuous response variables (EIT, spirometry, VCap, ΔPoes, and blood gas variables) had a normal distribution based on failure to reject the null hypothesis of normality using the Shapiro-Wilk test, and from visual inspection of Q-Q plots. Consequently, continuous data summarized as mean and 95% confidence interval.

Initial exploration of continuous responses used a mixed effect, linear model, including the random variance of horse and the fixed effect of order (first vs. second), time-point (T15, T30, T45, T60), and treatment (MASK vs. ETT), and the interactions. Significant effects in the model were determined at *p* < 0.05. There was no effect of order or time-point (T15 vs. T45 or T30 vs. T60), therefore further examination of the data excluded these from the model. Thus, data for each treatment were pooled, regardless of occurrence (first or second anesthesia) to result in data for six horses across two treatments (ETT, MASK) and two measurements (1, that included T15 and T45; and 2, that included T30 and T60) with two replicates. Each response was then averaged across replicates and tested using a mixed effect linear model including the random variance of the horse, the fixed effect of treatment (ETT, MASK), time (1, 2), and the interaction. When there was significant interaction at *p* < 0.05, *post-hoc*, paired comparisons were made across treatment and time against a Tukey-adjusted *p* < 0.05.

Global BrP was explored for an association with treatment and time using Cochran-Mantel-Haenszel analysis for stratified frequency data. To account for replication, the data was re-categorized with three responses for breathing patterns across replicates (normal:normal, alternate:alternate, normal: alternate, or alternate:normal). A significant general association was considered where the Chi-Square test statistic had a *p* < 0.05. The association of gBrP with PaO_2_ and with PaCO_2_ was explored using multinomial logistic regression with a significant association determined when the generated 95% confidence interval of the odds ratio excluded 1.0.

All statistical analyses were performed using SAS v 9.4 (SAS Institute, Cary, NC). Observations from endoscopic scoring of the larynx, frequency of oxygen supplementation (limited to the second trial within an anesthesia), and characteristics of Bnorm, Bsigh, and Bcrown patterns are described.

## Results

All six horses underwent and recovered from general anesthesia without complication and data was recorded at all time points required. One horse with MASK received additional xylazine and ketamine 10 min before T15.

### Continuous Variables

Continuous EIT variables are summarized as mean and 95% confidence interval (CI) (Table 1). Inspiratory to expiratory time ratio was smaller with ETT (0.017). The CoV_RL_ was significantly higher (i.e., shifted toward the non-dependent lung) during ETT compared to MASK (*p* = 0.025) but CoV_VD_ was not different. The ΔZ_R_ was significantly lower and ΔZ_L_ higher during ETT (*p* = 0.048). NSS (*p* = 0.032), but not DSS, was significantly greater with ETT. Tidal impedance variation (TIV) was significantly smaller in ETT (*p* = 0.048).

**Table 1:** Mean and 95% confidence internal (CI 95%) of continuous variables extracted from EIT.

**Treatment**	**MASK**				**ETT**			
**Time**	**1 (T15** **+** **45)**		**2 (T30** **+** **60)**		**1 (T15** **+** **45)**		**2 (T30** **+** **60)**	
	**Mean**	**CI 95%**	**Mean**	**CI 95%**	**Mean**	**CI 95%**	**Mean**	**CI 95%**
RR (min^−1^)	9.4^a^	3.6–15.2	11.0^a^	5.9–16.2	11.5^a^	6.2–16.9	13.2^a^	7.5–18.9
t_i_ (s)	3.0^a^	2.1–3.9	2.4^a^	1.8–2.9	2.3^a^	1.5–3.1	2.1^a^	1.4–2.8
t_e_ (s)	5.2^a^	2.9–7.5	3.9^a^	2.5–5.2	4.2^a^	1.4–7.0	3.4^a^	1.5–5.4
I:E ratio (1:x)*	1.5^a^	1.1–2.2	1.5^a^	1.3–2.0	0.9^a^	0.7–1.6	1.1^a^	0.8–1.9
CoV_VD_ (%)	49.5^a^	45.6–53.4	50.0^a^	46.5–53.5	48.9^a^	46.9–50.8	48.5^a^	45.2–51.8
CoV_RL_ (%)*	57.3^a^	54.4–60.2	58.1^ab^	55.1–61.2	59.0^ab^	56.4–61.6	61.6^b^	58.9–64.2
ΔZ_R_ (%)*	27^a^	22–33	25^ab^	19–31	23^ab^	19–28	18^b^	13–22
ΔZ_L_ (%)*	72.78^a^	67–78	75^ab^	69–81	77^ab^	72–81	82^b^	78–87
ΔZ_R_V__ (%)	−2^a^	−3–(−1)	−2^a^	−3–(−1)	−2^a^	−3–(−1)	−3^a^	−4–(−2)
ΔZ_R_CV__ (%)	8^a^	6–10	7^a^	5–9	7^a^	5–9	5^a^	3–8
ΔZ_R_CD__ (%)*	14^a^	13–16	14^a^	12–16	13^ab^	12–14	11^b^	9–12
ΔZ_R_D__ (%)	7^a^	4–10	7^a^	3–11	6^a^	4–8	5^a^	2–7
ΔZ_L_V__ (%)	9^a^	7–12	10^a^	7–13	11^a^	9–12	11^a^	9–12
ΔZ_L_CV__ (%)	27^a^	22–31	27^a^	23–31	28^a^	26–30	31^a^	26–35
ΔZ_L_CD__ (%)*	26^a^	24–28	27^ab^	24–29	27^ab^	25–29	30^b^	27–33
ΔZ_R_D__ (%)	11^a^	7–15	12^a^	10–13	11^a^	10–13	11^a^	4–17
NSS (%)*	19.1^a^	15.8–22.4	19.6^ab^	17.7–21.6	20.1^ab^	18.2–22.0	23.4^b^	20.2–26.6
DSS (%)	9.1^a^	4.3–13.9	8.5^a^	3.8–13.1	10.2^a^	7.2–13.2	12.1^a^	7.4–16.9
TIV (AU)*	5.37^a^	3.67–7.07	4.76^a^	3.40–6.12	4.09^a^	2.69–5.49	3.98^a^	2.71–5.25

RR, respiratory rate; ti, inspiratory time; te, expiratory time; I: E ratio, inspiratory to expiratory time ratio; CoV_VD_ and CoV_RL_, center of ventilation ventral to dorsal and right to left, respectively; ΔZ_R_ and ΔZ_L_: proportion of change in impedance within the right or left lung, respectively; ΔZ_R_V__, ΔZ_R_CV__, ΔZ_R_CD__, ΔZ_R_D__, ΔZ_L_V__, ΔZ_L_CV__, ΔZ_L_CD__ and ΔZ_L_D__: proportion of change in impedance within right and left (ventral, central-ventral, central-dorsal, dorsal) regions. NSS: non-dependent silent space; DSS, dependent silent space; TIV, tidal impedance variation (arbitrary units). *Variables with a significant global treatment effect in the mixed effect, linear model (p < 0.05). Means sharing the same superscript (^a, b^) within a row were not significantly different (Tukey adjusted p > 0.05).

Continuous variables relative to respiratory mechanics and gas exchange variables are summarized as mean and 95% CI (Table 2). Spirometry and VCap data from 10 out of 48 datasets had to be excluded due to Bcrown causing miscalculations by the spirometry software. For spirometry variables tested, only PEF (*p* = 0.009) and V_Dphys_/V_T_ (*p* = 0.034) were higher in ETT. The ΔPoes were significantly lower in ETT (*p* < 0.001). No significant treatment effect was found for PaO_2_, PaCO_2_, or ${\dot{\text{Q}}}_{\text{s}}/{\dot{\text{Q}}}_{\text{t}}$.

**Table 2:** Mean and 95% confidence interval (CI 95%) of continuous variables relative to respiratory mechanics and gas exchange.

**Treatment**	**MASK**				**ETT**			
**Time**	**1 (T15** **+** **45)**		**2 (T30** **+** **60)**		**1 (T15** **+** **45)**		**2 (T30** **+** **60)**	
	**Mean**	**CI 95%**	**Mean**	**CI 95%**	**Mean**	**CI 95%**	**Mean**	**CI 95%**
V_T_·kg^−1^ (ml·kg^−1^)	12.1^a^	9.2–14.9	11.4^a^	8.9–13.8	10.3^a^	5.4–15.3	8.9^a^	6.1–11.8
V_Talv_·kg^−1^ (ml·kg^−1^)	6.6^a^	3.8–9.3	6.5^a^	4.8–8.2	6.1^a^	1.7–10.5	4.5^a^	2.0–7.1
MV·kg^−1^ (ml·kg^−1^·min^−1^)	88^a^	60–117	104^a^	82–126	93^a^	58–128	103^a^	74–133
PIF (L/s)	47.6^a^	38.2–57.1	48.9^a^	41.9–55.8	51.4^a^	41.9–60.9	51.6^a^	47.8–55.4
PEF (L/s)*	84.8^a^	45.9–123.7	82.9^a^	51.1–114.6	126.1^a^	89.4–162.7	117.7^a^	90.8–144.7
EtCO_2_ (mmHg)	54.9^a^	48.3–61.7	52.5^a^	45.4–59.7	56.7^a^	49.6–63.8	55.1^a^	46.9–63.2
VCO_2_·kg^−1^	2.0^a^	1.6–2.3	2.4^a^	2.0–2.8	2.0^a^	1.2–2.9	2.4^a^	2.1–2.7
V_Daw_/V_T_	0.51^a^	0.37–0.64	0.47^a^	0.39–0.56	0.49^a^	0.30–0.67	0.56^a^	0.42–0.69
V_Dphys_/V_T_*	0.51^a^	0.42–0.60	0.53^a^	0.47–0.59	0.60^a^	0.21–1.00	0.64^a^	0.52–0.77
ΔPoes (cmH20)*	22.7^a^	14.1–31.2	22.9^a^	17.0–28.8	11.3^b^	7.8–14.8	11.5^b^	8.8–14.2
PaO_2_ (mmHg)	60.6^a^	56.3–64.8	66.0^a^	56.3–75.7	59.1^a^	51.3–67.0	59.5^a^	51.8–67.2
PaCO_2_ (mmHg)	58.8^a^	51.9–65.7	59.5^a^	53.4–65.5	55.6^a^	48.0–63.1	54.9^a^	46.5–63.3
${\dot{\text{Q}}}_{\text{s}}/{\dot{\text{Q}}}_{\text{t}}$	0.33^a^	0.27–0.38	0.34^a^	0.28–0.40	0.35^a^	0.30–0.39	0.36^a^	0.29–0.43

V_T_·kg^−1^, indexed tidal volume; V_Talv_·kg^−1^, indexed alveolar tidal volume; MV·kg^−1^, indexed minute ventilation; PIF and PEF, peak inspiratory and expiratory flow, respectively; EtCO_2_, end-tidal partial pressure of CO_2_; VCO_2_·kg^−1^, indexed elimination of CO_2_; V_Daw_/V_T_, airway dead space; V_Dphys_/V_T_, physiologic dead space; ΔPoes, esophageal pressure difference; PaO_2_, arterial partial pressure of oxygen; PaCO_2_, arterial partial pressure of carbon dioxide; ${\dot{\text{Q}}}_{\text{s}}/{\dot{\text{Q}}}_{\text{t}}$, venous admixture.^†^Means and CI 95% were calculated from 38/48 timepoints due to data exclusion. *Variables with a significant global treatment effect in the mixed effect, linear model (p < 0.05). Means sharing the same superscript (^a, b^) within a row were not significantly different (Tukey adjusted p > 0.05).

### Global Breathing Pattern

Global breathing pattern was significantly associated with treatment (*p* = 0.0121) but not time, with more alternate breaths (*sighs* and *crown-like*) during ETT (Table 3). There was no significant association of gBrP and PaO_2_ (OR 1.06, 95% CI 0.94–1.19) or PaCO_2_ (OR = 1.06, 95% CI 0.94–1.20).

**Table 3:** Frequencies of global breathing patterns (gBrP) in six horses (*n* = 6) anesthetized twice.

**Treatment**	**Time**	**gBrP N: N**	**gBrP Alt: Alt**	**gBrP N: Alt or Alt: N**	***N***
MASK	1 (T15 + 45)	3	3	0	6
	2 (T30 + 60)	3	3	0	6
ETT	1 (T15 + 45)	0	4	2	6
	2 (T30 + 60)	0	5	1	6

*Results for gBrP from each anesthesia (first: second) were collapsed into N: N (for the same horse, treatment and time only normal gBrP was observed in both anesthesias), Alt: Alt (for the same horse, treatment and time only alternate gBrP was observed in both anesthesias) and N: Alt or Alt: N (for the same horse, treatment and time different gBrP were observed in each anesthesia). Controlling for time, there was a significant association of treatment with gBrP (p = 0.0121). Controlling for treatment, there was no significant association of effect of time with gBrP (p = 0.8157)*.

### Descriptive Data

Two horses received oxygen supplementation after T30 during both anesthesias while two horses never required oxygen supplementation. The remaining two horses only received oxygen supplementation during ETT-MASK but not during MASK-ETT (one horse during the first and one during the second anesthesia). Thus, four of six (66%) horses required oxygen supplementation after T30 during ETT-MASK and two of six (33%) horses required oxygen supplementation after T30 during MASK-ETT.

**Figure 5:**
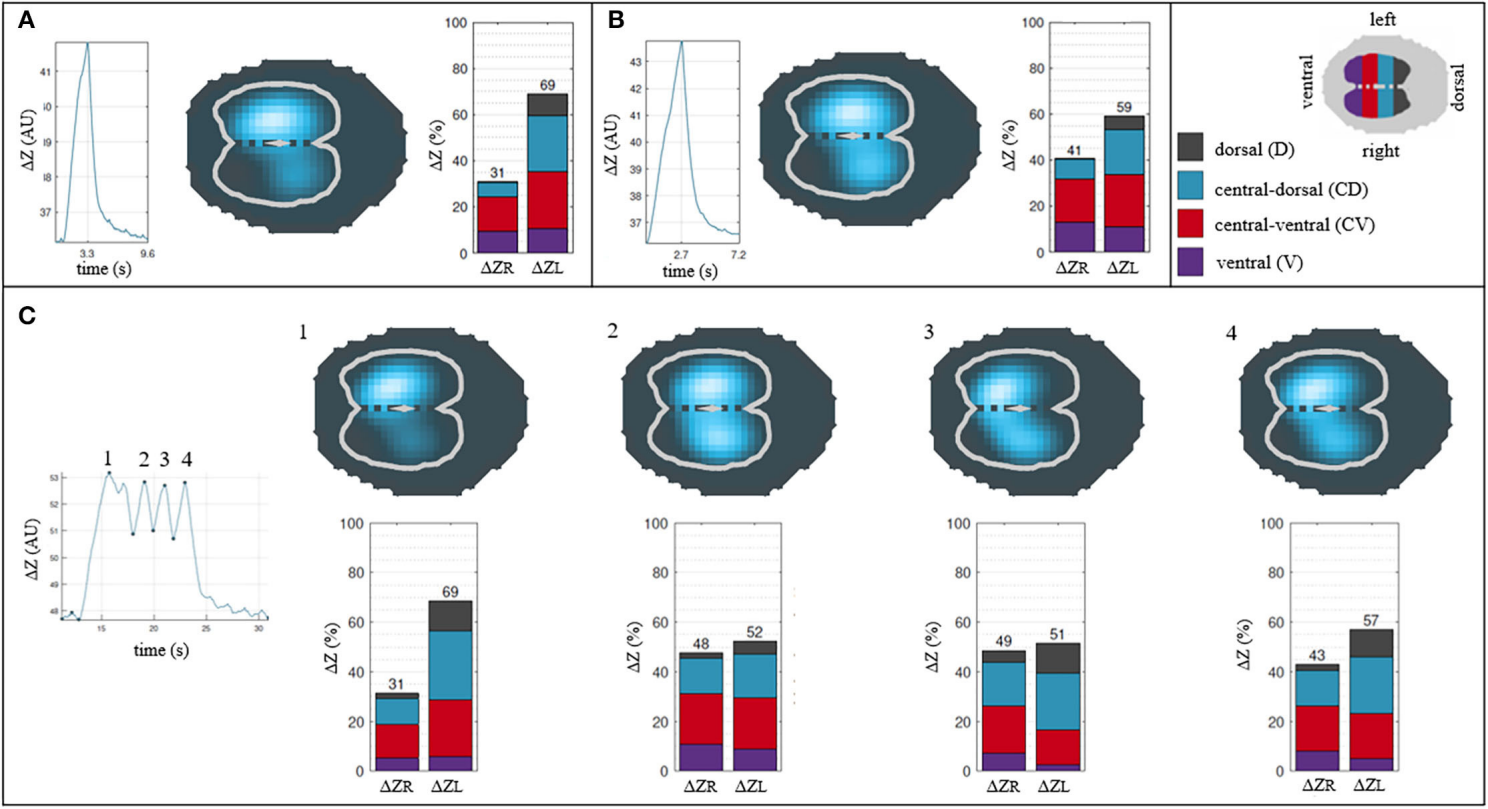
Descriptive analysis of the breath classification and their distribution of ventilation. **(A)** Normal breath. **(B)** *Sigh* breath. **(C)** *Crown-like* breath. Each breath is described by its impedance curve, a 2D representation of the EIT image* and a bar graph showing percentages of ΔZ for right and left lung (ΔZ_R_ and ΔZ_L_) and their respective 4 regions of interest (ΔZ_R_D__, ΔZ_R_CD__, ΔZ_R_CV__, ΔZ_R_V__ and ΔZ_L_D__, ΔZ_L_CD__, ΔZ_L_CV__, ΔZ_L_V__). Note that a *sigh* breath and *crown-like* breaths show a greater impedance change (blue area) in dependent regions of the lung compared to a normal breath. *cross-sectional reconstruction of the thorax with the two lung areas highlighted as white lines: the lighter blue, the greater the change in impedance of the area over one breath; darker blue denotes areas where minimal to no changes in impedance happened over one breath. Greater change in impedance implies higher ventilation in the corresponding lung area.

Five out of six horses showed Bcrown with two also having infrequent Bsigh. Only one horse did not show any *crown-like* breaths during all measurement periods. When present, Bsigh was observed occasionally interrupting runs of Bnorm. When present, Bcrown were mostly found in clusters (from two up to six breaths) at regular, repeated intervals. The observations around the distribution of ventilation for the Bnorm, Bsigh, and Bcrown are presented in Figure 5.

All horses with MASK showed partial adduction of the arytenoid cartilages during expiration (score = 2) in the endoscopic examination.

## Discussion

This study confirmed our hypothesis that breathing patterns would differ in anesthetized horses breathing via a mask or endotracheal tube with an increased frequency of *sighs* or *crown-like* breaths noted while the ETT was present. Furthermore, less ventilation was found in the dependent lung with an endotracheal tube compared to a facemask. The differences in peak expiratory flow, physiologic dead space, esophageal pressure difference, and I: E ratio noted in our study might explain these phenomena.

Regulation of the breathing pattern in mammals is complex and not fully understood (22). Under general anesthesia, multiple factors likely play a role in the regulation of the breath pattern such as acid-base status, hypoxemia, anesthetic protocol, and sensitive input from airway, lung, and muscle stretch receptors (22). Additionally, the complexity of the equine unique biphasic pattern poses further challenges for scientific evaluation and understanding (1, 2).

While the respiratory center in the pons is responsible for the rhythmogenesis of breathing, breathing pattern generation is more complex, with upper and lower airways being involved by modulating resistance to flow (9, 22). The presence of an endotracheal tube reduces airway resistance by bypassing the larynx, pharynx, and nasal cavity, which yield the highest resistance in the respiratory system (8, 23). In the present study, the horses' respiratory mechanics were significantly different during intubation with a smaller I:E ratio, higher peak expiratory flow, and lower esophageal pressure difference. A longer expiration together with a fast expiratory flow suggests a reduction in the time constant of the respiratory system, which is the main mechanism in adult mammals to maintain functional residual capacity (9). Thus, the presence of an endotracheal tube with its consequent drop in functional residual capacity could explain the differences in global breathing patterns between treatments (24, 25). Possibly, horses triggered alternate breaths more frequently with an endotracheal tube to compensate for the loss of functional residual capacity since *sighs* are known to act as natural recruitment maneuvers (25).

Hypercapnia and hypoxemia can trigger special breathing patterns like inspiratory breath holding in horses or *sigh* breaths in several species (4, 26–28). However, no association between arterial blood gases and alternate breaths was found in the study described here. Nevertheless, it is possible that hypoxemia or hypercapnia still contributed to triggering alternate breaths to counteract these gas exchange disturbances.

Based on the grouping of the different breath patterns within time points, *crown-like* breaths subjectively formed a more repeatable, organized pattern when compared to sighs, which were occasional alternate breaths. Furthermore, from the authors' observations, individual horses appeared to be more likely to show specific alternate breaths. One of the six horses only showed infrequent *sighs* as alternate breaths while the other five horses exhibited at least one *crown-like* breath during one of the measurement periods with two horses also having infrequent *sighs*.

The *crown-like* breath pattern observed in our horses does not match any of the typically defined breathing patterns in mammals (29). In intubated ponies, a similar breath pattern to the *crown-like* pattern was observed when anesthesia was maintained with isoflurane using respiratory ultrasonic plethysmography (RUP) (7). The abdominal circumference in RUP indicated incomplete expiration between several breaths before returning to baseline values. A regular polyphasic inspiratory flow was also described in endotracheally intubated horses anesthetized with isoflurane over 5 h (6). Interestingly in both studies, the *crown-like* breaths were observed during isoflurane maintenance while our horses displayed *crown-like* breaths on TIVA with xylazine, ketamine, and guaiphenesin. In the aforementioned study in ponies, intermittent deep breaths were observed during anesthesia when maintained with romifidine, ketamine, and midazolam (7). These deep breaths are comparable with the *sighs* observed in the horses in this study anesthetized with TIVA but using xylazine instead of romifidine, and guaiphenesin instead of midazolam. Thus, it appears that the presence of alternate breaths is not specific to any one anesthetic protocol.

Endotracheal intubation in equine anesthesia provides a secured airway and allows the clinician to apply controlled ventilation to counteract hypoventilation. However, since anesthetized healthy horses exhibit no to mild hypoventilation when on *triple drip* (30) and will rarely regurgitate and aspirate gastrointestinal contents, the effects of an endotracheal tube during spontaneous ventilation are worth further research.

It was hypothesized that the presence of an endotracheal tube would affect the distribution of ventilation, which was confirmed by noting an overall shift of the center of ventilation from the right (dependent) lung toward the left (non-dependent) lung when horses were breathing through an endotracheal tube. The higher ΔPoes while breathing through a mask indicates greater transpulmonary pressures recruiting dependent parts of the lungs at a more distensible point within the compliance curve of the lungs (31). Moreover, longer inspiratory times in relation to expiratory times while breathing through a mask may allow lung units with longer time constants in the dependent lung to be recruited causing the change in ventilation toward the dependent lung (31). This shift was expected to decrease venous admixture. However, a difference in the venous admixture was not found between treatments. One explanation would be that the increase in ventilation in the dependent lung was insufficient for any effect on a gas exchange or that the effect was not detectable with only six horses. Alternately, the *crown-like* breaths observed while intubated could be a strategy aiming to partially compensate and shift ventilation toward the dependent lung for some of the low volume breaths (Figure 3). Although *crown-like* breaths were insufficient to counteract the significant difference we found between treatments for the distribution of ventilation, they could have prevented a significant difference in venous admixture by increasing the surface area of the lung sufficiently (32).

Despite ventilation shifting toward the non-dependent left lung, the higher NSS seen with an endotracheal tube indicates that more non-dependent lung units in the lung periphery were hypoventilated when compared to facemask breathing. The lower tidal impedance variation while intubated supports this finding as lower tidal volumes lead to more ventilation in the hilar area of the lungs (33). Following the West zones (34) and the *slinky spring* model (35, 36) these peripheral lung units are open although hypoventilated. This also explains our finding of a significantly higher physiological dead space with an endotracheal tube. With no difference in airway dead space for the two treatments, we assume that a higher proportion of alveolar dead space occurred during the intubated periods, which means that more lung units received relatively more ventilation than perfusion (high V/Q mismatch) during this treatment. The main reason for an increase of alveolar dead space during anesthesia is overdistension of lung units due to positive pressure ventilation. Since the horses were spontaneously breathing, overdistension of lung units as a cause for the increase in alveolar dead space can be excluded (37), allowing the conclusion that the lower perfusion was either caused by low cardiac output and/or redistribution of blood away from hypoventilated lung units due to the maintained hypoxic pulmonary vasoconstriction during TIVA (38).

Although tidal impedance variation has been shown to have a direct linear relationship with tidal volume, we found significantly greater TIV with a facemask, while there was no difference in V_T_ between treatments. This conflict in results can be explained by measurement issues due to spirometry software being unable to analyze the *crown-like* breaths. However, we were able to adapt the breath detection algorithm with the EIT. This allowed a complete dataset for TIV to be statistically analyzed, while >20% of V_T_ measurements had to be excluded.

After observations in horses on treadmills, during swimming, and in anesthetized ponies maintained on volatile anesthetics (3, 5, 39), it was expected that significant expiratory laryngeal adduction would occur during mask breathing. However, the horses in this study only showed partial expiratory laryngeal adduction during mask breathing. This points toward diaphragmatic involvement as the main influence of breathing patterns in our horses when laryngeal braking is not possible while intubated (26).

The main limitation of this study is the small number of horses, which is common for experimental equine anesthesia studies due to the high anesthetic risk for the individual horse and cost. To compensate for the low number, we decided to use a cross over design with all horses undergoing both treatments twice, in randomized order. This resulted in two replicates for each horse in each treatment, given the order had no effect, thereby maximizing the information from a small sample size. For safety reasons, only a *triple drip* anesthesia protocol, common for non-intubated horses in the field, was used to avoid inhalant exposure to the researchers. This created a relatively short observational period by purposely avoiding prolonged anesthesia using triple drip (40). This time frame was, however, considered long enough to note changes in the distribution of ventilation (33).

Future studies will need to use a bigger sample size to evaluate more in detail factors that could be affecting breathing patterns in anesthetized horses including anesthesia duration and protocol, hypercapnia, hypoxemia, airway management, and individuality of the horse or pony. Of especial interest would be the impact of these factors on gas exchange and distribution of ventilation during the recovery period, when most equine anesthesia fatalities occur.

In conclusion, endotracheal intubation leads to alternate breathing patterns including *sighs* and *crown-like* breaths, and shifts the ventilation toward the non-dependent parts of the lungs in horses anesthetized with xylazine, ketamine, and guaiphenesin. While short anesthesia without endotracheal intubation could have a beneficial effect on the distribution of ventilation, no impact on gas exchange was observed between treatments, and therefore, the clinical relevance of these observations is unclear.

## Data Availability Statement

The original contributions presented in the study are included in the article/supplementary material, further inquiries can be directed to the corresponding author/s.

## Ethics Statement

The animal study was reviewed and approved by Animal Ethics Committee at Murdoch University.

## Author Contributions

FM-M and MM: study design, data collection, data analysis, manuscript preparation, and final approval. DB and AR: data collection, manuscript preparation, and final approval. GH: data analysis (statistics), manuscript preparation, and final approval. AW: data analysis, manuscript preparation, and final approval. All authors contributed to the article and approved the submitted version.

## Funding

This research project was supported and financed by the SVM funding from Murdoch University.

## Conflict of Interest

The authors declare that the research was conducted in the absence of any commercial or financial relationships that could be construed as a potential conflict of interest.

## Publisher's Note

All claims expressed in this article are solely those of the authors and do not necessarily represent those of their affiliated organizations, or those of the publisher, the editors and the reviewers. Any product that may be evaluated in this article, or claim that may be made by its manufacturer, is not guaranteed or endorsed by the publisher.
